# A Nanoporous Alumina Membrane Based Electrochemical Biosensor for Histamine Determination with Biofunctionalized Magnetic Nanoparticles Concentration and Signal Amplification

**DOI:** 10.3390/s16101767

**Published:** 2016-10-22

**Authors:** Weiwei Ye, Yifan Xu, Lihao Zheng, Yu Zhang, Mo Yang, Peilong Sun

**Affiliations:** 1 Institute of Ocean Research, Zhejiang University of Technology, Hangzhou 310014, China; 2 Department of Food Science and Technology, Zhejiang University of Technology, Hangzhou 310014, China; Lvanwork@163.com (Y.X.); zhenlihaoll.2008@163.com (L.Z.); zhangyuzjut@163.com (Y.Z.); sun_pl@zjut.edu.cn (P.S.); 3 Interdisciplinary Division of Biomedical Engineering, The Hong Kong Polytechnic University, HungHom, Kowloon, Hong Kong, China; mo.yang@polyu.edu.hk

**Keywords:** nanoporous alumina membrane, histamine, magnetic nanoparticles, electrochemical biosensor

## Abstract

Histamine is an indicator of food quality and indispensable in the efficient functioning of various physiological systems. Rapid and sensitive determination of histamine is urgently needed in food analysis and clinical diagnostics. Traditional histamine detection methods require qualified personnel, need complex operation processes, and are time-consuming. In this study, a biofunctionalized nanoporous alumina membrane based electrochemical biosensor with magnetic nanoparticles (MNPs) concentration and signal amplification was developed for histamine determination. Nanoporous alumina membranes were modified by anti-histamine antibody and integrated into polydimethylsiloxane (PDMS) chambers. The specific antibody modified MNPs were used to concentrate histamine from samples and transferred to the antibody modified nanoporous membrane. The MNPs conjugated to histamine were captured in the nanopores via specific reaction between histamine and anti-histamine antibody, resulting in a blocking effect that was amplified by MNPs in the nanopores. The blockage signals could be measured by electrochemical impedance spectroscopy across the nanoporous alumina membrane. The sensing platform had great sensitivity and the limit of detection (LOD) reached as low as 3 nM. This biosensor could be successfully applied for histamine determination in saury that was stored in frozen conditions for different hours, presenting a potentially novel, sensitive, and specific sensing system for food quality assessment and safety support.

## 1. Introduction

Histamine, one of the biogenic amines, is synthesized by basophils, mast cells, enterochromaffine cells, platelets, and histaminergic neurons in vivo. It is stored in vesicles and released on stimulation. Histamine is also formed by enzymatic decarbonylation of histidine by bacterial action with improper processing procedures or inappropriate storage conditions of food, and hence could be present in substantial amounts in fermented food stuffs and seafood, especially saury, mackerel, sardine, tuna, herring, and anchovy [1,2]. Therefore, it is often used as one of the indicators for food quality control in the processes of production, storage, transportation, and transaction [3]. Histamine can enter the human body by dietary sources and can bind to receptors on cellular membranes in the gastrointestinal, cardiovascular, and haemotological immunological system, thereby initiating allergic reactions such as hypotension, flushing, diarrhea, vomiting, and headaches [4,5]. The symptoms may vary between individuals who are exposed to the same level of histamine in contaminated food [6]. High levels of histamine can even cause cancer [7]. Thus, rapid and sensitive determination of histamine is urgently needed in clinical and food analysis. 

The current histamine detection methods include high-performance liquid chromatography (HPLC), chromatographic analysis, and capillary zone electrophoresis [8,9,10]. These methods for histamine analysis often require qualified personnel, extensive sample cleanup, and derivatization procedures. Immuno-enzymatic techniques such as enzyme-linked immunosorbent assay (ELISA) are usually used to perform screening for histamine poisoning, allowing a more rapid analysis method with less operation procedures being required and offering a quick screening method for industrial food quality evaluation, although the method is qualitative or semi-quantitative [11]. In recent years, miniaturized and integrated biosensors have been developed for direct, fast, specific, and sensitive histamine detection in seafood spoilage [12,13,14,15]. Various biosensors have been explored for histamine detection, such as fluorescence biosensors, molecularly imprinted polymer (MIP) histamine sensors, and electrochemical histamine sensors [16,17,18,19,20,21,22]. Among these methods, MIP and electrochemical histamine sensors could provide rapid histamine determination methods with low costs. MIP histamine sensors are pH dependent, limiting their wide application in clinical diagnostics and food analysis. Electrochemical biosensors are inexpensive techniques for histamine binding event detection by monitoring electrochemical spectra. Metal electrodes are used in most of the currently developed electrochemical sensing platforms. The electrode polarization effect limits the performance and application of these sensors [23]. 

Compared to flat substrates, nanostructured substrates with through nanopores have large surface affinity areas and allow ions to pass through. With the ability of integrating electrochemical impedance spectroscopy, nanostructured substrates have been applied for biosensing across a wide range. Nanoporous alumina membranes have become a hot research area because of their established fabrication processes, nano-ordered structures, dramatically large surface areas, easy surface modifications, high surface reaction rate, as well as their intensified output signals [24]. Wide applications of nanoporous alumina membranes are in areas of DNA detection, lipid membrane-based biosensors, foodborne pathogen biosensing, and virus and cancer cell detection [25,26,27,28,29,30,31]. However, the application of nanoporous alumina membranes for histamine determination has not yet been explored. Due to the easy surface modification and non-conductive frame with through nanopores, nanoporous alumina membranes are suitable candidates for constructing electrochemical sensing platforms for histamine investigation. 

The sensing mechanism for histamine study is based on the blocking effect of molecules in the nanopores. However, it is quite challenging for small histamine molecules to block nanopores with large efficiency. Nanoparticles are needed as tags to increase the blocking effect and amplify signals. In our previous study, a gold nanoparticle label based sensing platform was developed for sensing DNA with great blockage efficiency and detection sensitivity [32]. Magnetic nanoparticles (MNPs) can be easily modified for biomolecule capture and concentration under magnetism field [33]. Paramagnetic microparticles and nanoparticles with nanomaghemite cores were used to off-line couple with ion exchange liquid chromatography for isolation of biogenic amines and detection of histamine in silages, respectively [34,35]. MNPs with the diameter of about 5–20 nm have the ability of entering 100 nm nanopores [36,37]. These properties make MNPs good candidates for concentrating histamine from samples to nanoporous membranes and increasing the blocking effect in the nanopores as well. 

In this paper, we reported the use of nanoporous alumina membranes based electrochemical biosensor with biofunctional MNP concentration for histamine determination in seafood. Nanoporous alumina membranes were functionalized with anti-histamine antibody. Biofunctional MNPs concentrated histamine under the external magnetic field to conjugate in the nanopores. The application of MNPs brought enhanced detection sensitivity to the electrochemical biosensor, whose output signals were measured by the change of impedance spectra. This nanoporous alumina membrane based sensing platform with MNPs for concentrating target molecules and increasing blocking effect shows the ability for rapid and sensitive histamine determination in field.

## 2. Materials and Methods 

### 2.1. Materials

Nanoporous alumina membranes were purchased from Whatman, Inc., Maidstone, UK. The membranes were round with 13 mm diameter and were 60 μm thick. The nanopores were about 100 nm in diameter. Histamine and anti-histamine antibody produced in rabbits were purchased from Sigma Aldrich (St. Louis, MO, USA). (3-glycidoxypropyl) trimethoxysilane (GPMS, 98%), *N*-(3-Dimethylaminopropyl)-*N*-ethylcarbodiimide hydrochloride (EDC), *N*-Hydroxysuccinimide (NHS), trichloroacetic acid (99%), sodium hydroxide (98%), and n-butanol (99.8%) were also bought from Sigma Aldrich. Dimercaptosuccinic acid (DMSA) modified Fe_3_O_4_ MNPs were obtained from XFNANO (Nanjing, China). Toluene (99.8%), anhydrous ethanol (99%), and hydrogen peroxide (30%) were purchased from Sigma Aldrich. All of these chemicals were used as received without further purification.

### 2.2. Biofunctionalization of Nanoporous Alumina Membrane

**Figure 1:**
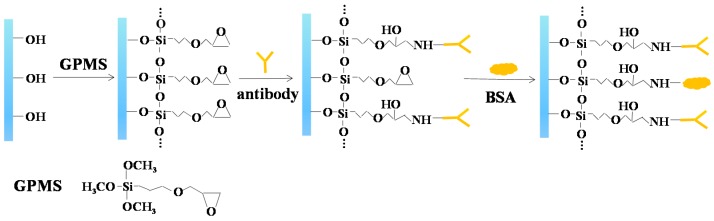
Scheme for surface modification of nanoporous alumina membrane by (3-glycidoxypropyl) trimethoxysilane (GPMS) and conjugation with anti-histamine antibody.

The biofunctionalization process of nanoporous alumina membranes was based on our previous study [38]. The scheme for surface modification of nanoporous alumina membrane by GPMS and anti-histamine antibody conjugation was shown in Figure 1. Nanoporous alumina membranes were kept in boiling hydrogen peroxide for 30 min to clean the membranes and hydroxyl groups formed on the nanopore surfaces. The membranes were immersed in deionized (DI) water on shakers for 15 min. The dried membranes were functionalized with the mixture of 2% GPMS and toluene at the temperature of 60 °C overnight, and alternatingly rinsed by toluene and anhydrous ethanol. After rinsing three times, the membranes were cured at 60 °C for 2 h to form a self-assembled monolayer on the nanopore surface. To immobilize anti-histamine antibody on silanized nanoporous membranes, antibody in phosphate buffer solution (PBS) buffer (pH 7.4) was injected into the nanoporous alumina membranes for coupling under 4 °C for about 4 h. The antibody was immobilized in the nanopores by chemical reaction between amino groups and epoxy groups. In addition, bovine serum albumin (BSA) was used as the blocking agent to reduce non-specific binding. The nanoporous alumina membranes were rinsed and then immersed in 1% BSA (*w/w*) in PBS (pH 8.0) for about 4 h. The biofunctionalized nanoporous membranes were then rinsed with PBS (pH 7.4) for later use.

### 2.3. Magnetic Nanoparticles Functionalization

Dimercaptosuccinic acid (DMSA) modified MNPs with an average diameter of about 10 nm were activated by EDC/NHS for anti-histamine antibody immobilization. MNPs (100 μL, 4 mg/mL) were contained in a tube and added with PBS (100 μL, 0.01 M, pH 6) for thorough mixing. A magnetic separator was placed under the tube which pulled MNPs to the bottom under the magnetic field. The supernatant was pipetted carefully out of the tube and nanoparticles were resuspended in PBS for rinsing. This was repeated for three times and the nanoparticles were then suspended in a 100 μL PBS (0.01 M, pH 6) solution. The rinsed DMSA modified MNPs (600 μL, 0.4 mg/mL) were activated by EDC/NHS [39]. EDC (0.26 M, 3 μL) and NHS (0.26 M, 1.5 μL) were added to MNPs (600 μL, 0.4 mg/mL) and mixed by vortex in a centrifuge tube (1.5 mL). After half an hour, the centrifuge tube was put on a magnetic separator to pull the activated nanoparticles to the bottom of the tube. The supernatant was pipetted out of the tube and the unreacted EDC/NHS was removed by magnetic field. Nanoparticles were rinsed and dispersed in PBS (600 μL, 0.01 M, pH 8.0). Antibody solution (12 μL, 0.73 mg/mL) was immediately added and mixed with the magnetic nanoparticles at room temperature for 4 h. Then, the antibody-nanoparticles conjugation was separated by magnetic field and the supernatant was pipetted out of the tube carefully. The conjugation was rinsed with PBS three times and dispersed in 1% BSA (w/w) in PBS (600 μL, 0.01 M, pH 8.0) for 4 h to block the unspecific binding sites. The biofunctionalized MNPs were rinsed with PBS (600 μL, 0.01 M, pH 7.4) and resuspended in the PBS solution for later use.

### 2.4. Histamine Extraction from Saury Fish

Fish samples of saury were purchased at the local market. They were cleaned with the giblets removed. Extraction was performed using 5 g of homogenized saury according to the previous study [4]. The flesh of the fish was directly weighed in 50 mL capped polyethylene centrifuge tubes and mixed with trichloroacetic acid solution (15 mL, 100 g/L). The mixture was sonicated for 30 min and filtered through a 0.45 mm membrane to obtain a 50 mL filtrate. Sodium hydroxide was added to the filtrate (2 mL) to make the solution alkaline, and n-butanol (3 mL) was also added. The mixture was mixed in a vortex mixer for 1 min. Histamine in n-butanol solution was obtained by separation. The procedure was repeated three times. The three extracts were gathered, adjusting the volume to a final 10 mL with n-butanol. The extracts in n-butanol (2 mL) were added with hydrochloric acid (3 mL, 3%) and mixed thoroughly. Histamine was extracted in hydrochloric acid by separation. The procedure was repeated five times and the extracts were gathered, adjusting the volume to a final 10 mL with hydrochloric acid (3%). Sodium hydroxide was used to adjust the pH of the extracts to pH 7.4.

### 2.5. Histamine Concentration to Biofunctionalized Nanoporous Alumina Membrane

Anti-histamine antibody modified MNPs (100 μL, 0.4 mg/mL) were mixed with the above histamine extracts (100 μL) for 30 min for conjugation. The MNPs-histamine conjugation was pulled to the tube bottom by magnetic field with supernatant removed and added to the antibody biofunctionalized nanoporous alumina membrane. The membrane was placed on a small permanent magnet and left for 30 min. Histamine conjugated with magnetic nanoparticles were concentrated on the membrane sensing surface and captured by the antibody. The membrane was rinsed with PBS three times to wash away the unbounded histamine-MNP conjugation.

### 2.6. Characterization

The morphology of the MNPs and antibody modified MNPs were characterized by JEOL-2100F transmission electron microscopy (TEM) equipped with an Oxford Instrument energy-dispersive X-ray (EDX) spectrometry system, operating at 200 kV. Samples (10 μL) were dropped on holey carbon coated 400 mesh copper grids and dried for TEM characterization. The hydrodynamic size and the Zeta potential were measured on a NanoBrook Omni Particle Sizer and Zeta Potential Analyzer (Brookhaven Instruments Corporation, Austin, TX, USA). The scan was conducted three times and the zeta potential values were averaged. Surface morphologies of bare nanoporous alumina membranes and functionalized nanoporous membranes with MNPs conjugation were characterized by scanning electron microscopy (SEM). The prepared dry samples were coated with gold for 60 s and imaged by a JEOL model JSM-6490 SEM system.

### 2.7. Electrochemical Impedance Spectroscopy Measurement

Electrochemical impedance spectroscopy was recorded by a two-electrodes system across the membrane. The polydimethylsiloxane (PDMS) chamber was fabricated by Sylgard 184 (Dow Corning, Midland, MI, USA) with the assistance of a mold. The prepared nanoporous alumina membrane was integrated into PDMS chamber, which was separated into an upper chamber and lower chamber. Two platinum (Pt) electrodes were inserted into the two chambers as the working electrode and reference electrode, respectively. The PDMS chambers were filled with PBS. The impedance measurement was performed on the electrochemical analyzer Autolab Potentiostat Galvanostat (Metroholm, Zofingen, Switzerland) with 50 mVpp voltage which was controlled by NOVA 1.10 software package (Metroholm, Zofingen, Switzerland).

## 3. Results and Discussion

### 3.1. Mechanism of Histamine Detection by Electrochemical Biosensor

**Figure 2:**
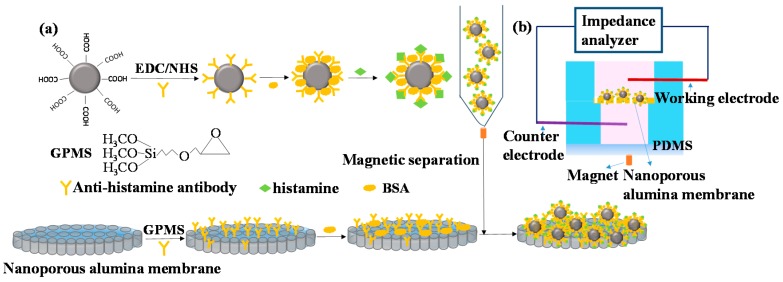
Schematic diagram of biofunctionalization of magnetic nanoparticles (MNPs) for histamine concentration to nanoporous alumina membrane (**a**) with electrochemical biosensing system for detection (**b**). EDC, *N*-(3-Dimethylaminopropyl)-*N*-ethylcarbodiimide hydrochloride; NHS, *N*-Hydroxysuccinimide; BSA, bovine serum albumin; PDMS, polydimethylsiloxane.

The sensing mechanism of the proposed nanoporous alumina membrane based electrochemical biosensor for histamine sensing assisted by MNP concentration is shown in Figure 2. DMSA modified Fe_3_O_4_ MNPs were activated by EDC/NHS and modified with anti-histamine antibody. When the biofunctionalized MNPs conjugated histamine from samples, they were concentrated and separated under magnetic field. Nanoporous alumina membranes were modified by GPMS and immobilized with anti-histamine antibody. BSA was used as a blocking agent to reduce non-specific binding of biomolecules. The membrane was anchored in the PDMS chamber. The MNPs-histamine conjugation was concentrated to the nanoporous membrane and captured in nanopores by the reaction between histamine and the anti-histamine antibody (Figure 2a). MNPs-histamine conjugation caused blocking effect in the nanopores that can be measured by impedance analyzer (Figure 2b). Two platinum electrodes were immersed in electrolyte solution in the upper and lower chamber for current measurement using an electrochemical impedance analyzer. For nanoporous membranes immobilized with antibody, ions passed through nanopores and large current was detected. The capture of target histamine conjugated MNPs increased the blocking effect in the nanopores leading to the decrease of the electrolyte current and increase in the impedance signals. By recording the impedance spectra across the membranes, histamine was detected. In this system, the measured current was mainly based on electrolyte ion flow in the solution through the nanopores. The main effect of MNPs was to concentrate histamine from samples and to amplify blocking of ion flow in the nanopores. 

### 3.2. Characterization of Magnetic Nanoparticles and Nanoporous Alumina Membranes

**Figure 3:**
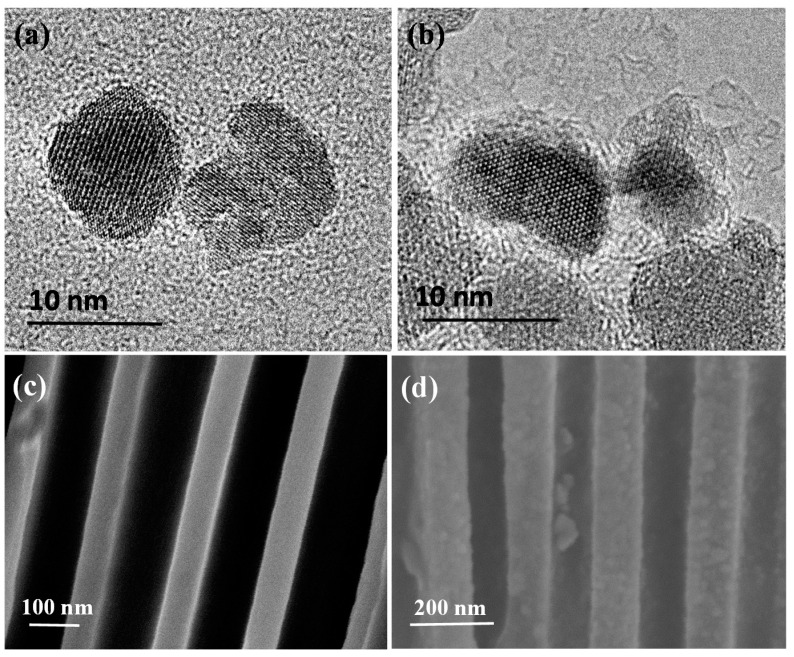
Transmission electron microscopy (TEM) images of magnetic nanoparticles (MNPs) (**a**) and antibody conjugated MNPs (**b**); scanning electron microscopy (SEM) images of cross-sectional view of bare nanoporous alumina membranes (**c**) and antibody functionalized nanoporous alumina membranes after histamine-MNPs conjugation capture (**d**).

DMSA modified MNPs were used to conjugate histamine molecules and concentrate histamine to the antibody modified nanoporous membrane, forming MNPs–histamine–nanoporous membrane sandwich structures in the nanopores. The size and morphology of MNPs and antibody conjugated MNPs were characterized by TEM. High resolution TEM (HRTEM) showed that the average diameter of MNPs was about 10 nm dispersed in DI water (Figure 3a). A thin layer of antibody was observed around the MNPs in Figure 3b. Zeta potential measurement showed that the average potential of nanoparticles shifted from −42.7 mV to −26.6 mV after antibody conjugation, which was mainly due to the charges of the conjugated anti-histamine antibody. This further confirmed successful conjugation of antibody on DMSA modified MNPs. The average hydrodynamic size was about 15 nm. The entrance of MNPs into nanopores in the nanoporous alumina membranes was affected by nanoparticle surface interaction and confinement effect [40]. Small sizes decrease the effect and make the entrance possible. SEM was applied to confirm the conjugation between nanoparticles and nanoporous membranes. SEM images showed cross-sectional views of bare nanoporous alumina membranes and nanoporous alumina membranes with histamine-MNPs conjugation. It could be observed that no blocking in the cross-sectional view of nanopores without target capture occurred (Figure 3c). When histamine conjugated MNPs were captured on antibody modified nanoporous alumina membranes, it could be observed that nanoparticle tags were attached on nanopore walls (Figure 3d). The formation of nanoparticle tags could lead to the blockage of ion flow across nanoporous alumina membranes.

### 3.3. Antibody Immobilization on Nanoporous Membrane

Various concentrations of anti-histamine antibody were immobilized on GPMS modified nanoporous alumina membranes and the impedance spectra were recorded to monitor the blocking effect in the nanopores caused by the antibody. During the experiment, PBS was used to gently rinse off unbounded species three times before impedance measuring. Impedance observably increased after antibody was immobilized on the nanoporous alumina membranes, with the greatest impedance increase rate at the concentration of 7 μg/mL. As antibody concentration increased, the impedance increase rate experienced no obvious change. This is because the functionalized sites of membrane and nanopore walls were conjugated with antibody and no more sites for further conjugation. Therefore, the measured impedance amplitude for antibody immobilization on nanoporous membrane reached the maximum. This explained why when the concentration of antibody increased, the impedance amplitude had no further increase. Therefore, the antibody concentration of 7 μg/mL was chosen for later experiment on histamine detection.

### 3.4. Histamine Detection without MNPs

**Figure 4:**
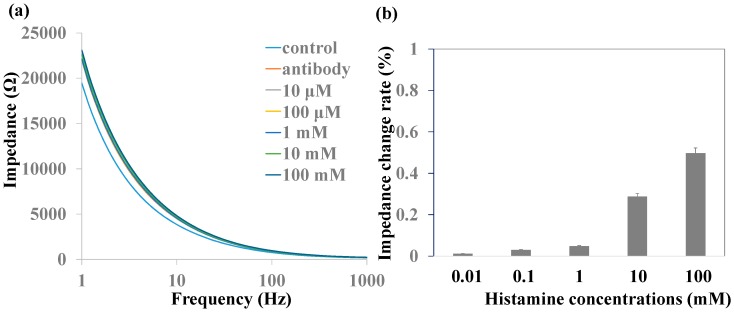
(**a**) Impedance spectra and (**b**) impedance change rate of various histamine concentrations without magnetic nanoparticles (MNPs).

Histamine detection without MNPs was measured by impedance spectra. Histamine with various concentrations was added into the chamber by a syringe pump and stayed for 30 min to be captured by anti-histamine antibody in the nanoporous alumina membranes. Next, PBS was added into the chamber gently to carry away the nonspecific adsorption of molecules on the nanoporous membranes three times before the impedance measurement. Figure 4a shows the impedance spectra of the biofunctionalized nanoporous alumina membrane and various concentrations of histamine capture without MNPs. Impedance increased after antibody immobilization and various concentrations of histamine capture on nanoporous alumina membranes. However, the impedance spectra had no obvious increase with the histamine concentrations from 10 μM to 100 mM. The relative impedance amplitude change is shown in Figure 4b. Compared to impedance value after antibody immobilization, impedance increased less than about 0.5% even with the large concentration of 100 mM because small histamine molecules did not produce an obvious blocking effect without the nanoparticles.

### 3.5. The Effect of MNPs on Histamine Detection

**Figure 5:**
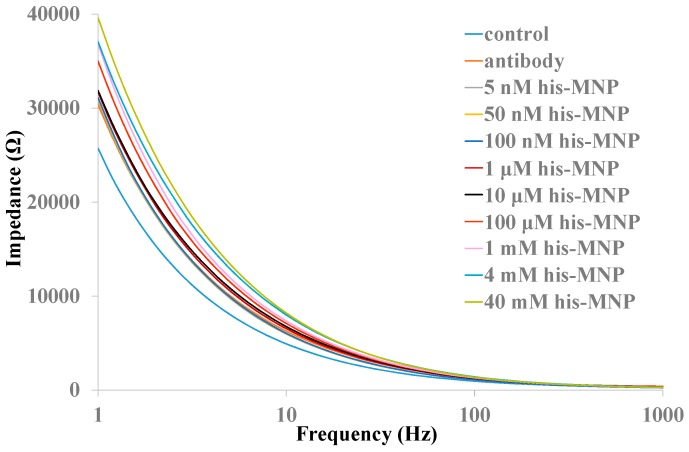
Impedance spectra of various histamine concentrations with MNPs in a nanoporous alumina membrane.

In order to increase detection sensitivity of the biosensor, anti-histamine antibody modified MNPs were used to concentrate histamine from samples. Histamine-MNPs conjugation was transferred to antibody modified nanoporous alumina membranes and remained for 30 min in order to make sure that the full reaction of histamine and antibody in nanopores occurred. Impedance spectroscopy was recorded across the nanoporous alumina membrane. Impedance increased obviously as histamine concentrations increased from 1 μM to 40 mM, with the results shown in Figure 5. The maximum impedance change appeared at a low frequency range, which could be described by an equivalent circuit model consisting of resistance in series with parallel connection of resistance and capacitance that was described in our previous study [38]. The impedance calculated from the model could be expressed by Equation (1).

(1) $$Z=\frac{R_{m}}{j2\pi fR_{m}C_{m}+1}+R_{el},$$

where *R_m_* and *C_m_* represent the resistance and capacitance of the nanoporous alumina membrane, respectively, *f* is the frequency, and *R_el_* is the electrolyte resistance between the two platinum electrodes. Impedance changes with frequency changes. When the frequency becomes low, impedance change increases.

**Figure 6:**
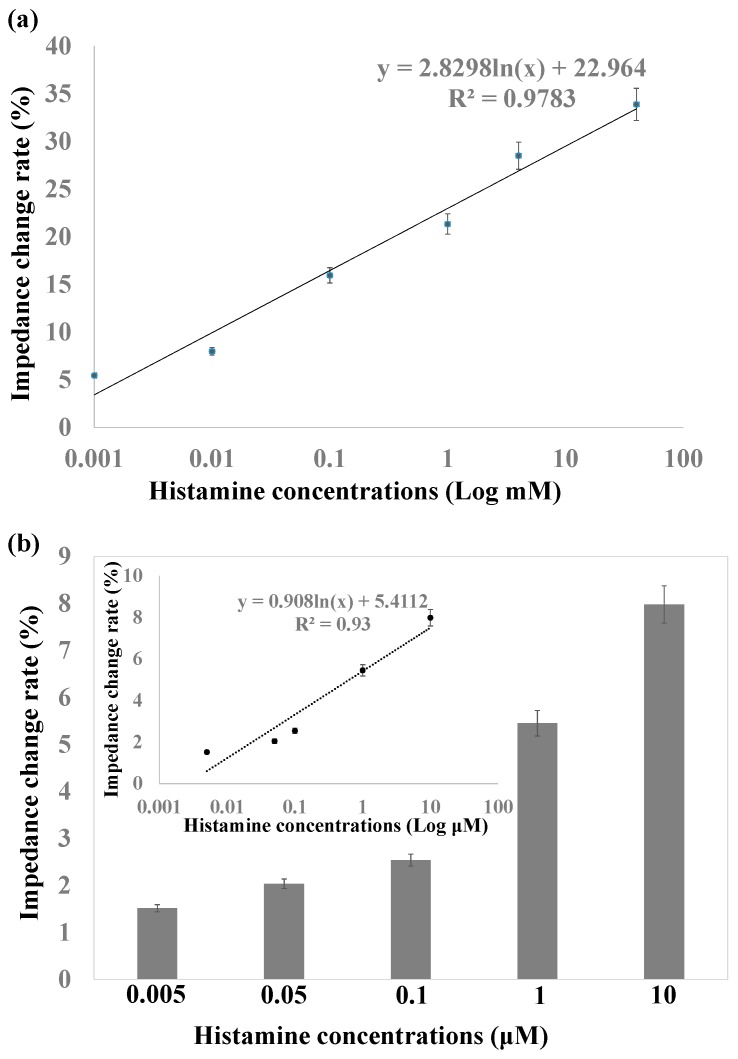
(**a**) Linear relationship between impedance change rate versus histamine concentrations from 1 μM to 100 mM; (**b**) Impedance change with histamine concentrations from 5 nM to 10 μM.

The relative impedance amplitude change rate of different histamine concentrations compared to antibody immobilized nanoporous alumina membranes is shown in Figure 6a. The change rate indicated rather good linearity with impedance increases from 5% to 33.9% for histamine concentrations from 1 μM to 40 mM. The detection sensitivity increased greatly compared to histamine detection by the biosensing system based on nanoporous alumina membrane without MNPs. This is due to the concentration of target histamine by MNPs, which also increased the blocking effect in the nanopores and therefore improved the sensitivity of detection. Impedance change with low concentrations of histamine from 5 nM to 10 μM is shown in Figure 6b. The impedance change rate had linearity with logarithmic histamine concentration shown in the inset figure of Figure 6. The limit of detection (LOD) was calculated by the control signal plus three times the noise signal (standard deviation). The LOD of this biosensor was as low as 3 nM for histamine detection. As the histamine concentrations ranged from tens of micromolars to hundreds of micromolars in rotted food at different spoilage stages, the sensing system showed the potential for sensitive histamine detection for safety support of fish and seafood products [41].

**Figure 7:**
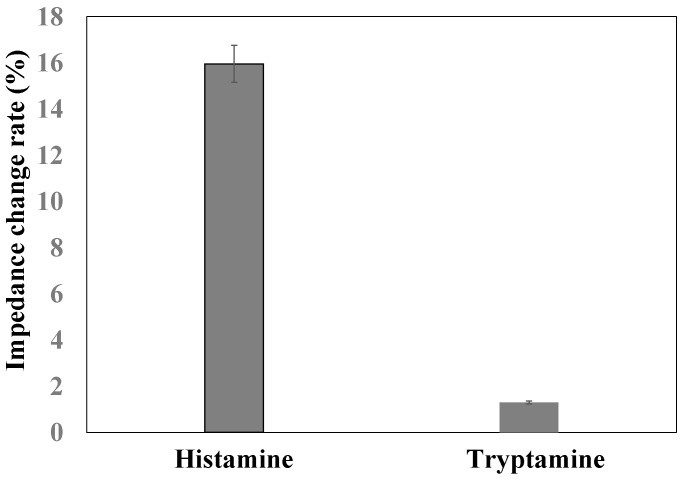
Comparison of impedance change rate of tryptamine and histamine detection.

In order to monitor the specificity of this nanoporous alumina membrane based electrochemical biosensor for histamine detection, tryptamine and histamine (100 μM) were measured with the same procedure. As shown in Figure 7, compared to impedance with antibody immobilization, the impedance increased about 1.3% and 15.3% for tryptamine and histamine detection, respectively. The impedance increase of target histamine capture was more than that of non-target tryptamine capture for the same concentration, which demonstrated good specificity of this nanoporous alumina membrane based electrochemical biosensor with MNPs conjugation and signal amplification for histamine detection.

### 3.6. Histamine Determination in Saury

To evaluate the ability of this electrochemistry biosensor to determine histamine concentrations in fish, histamine concentrations of saury samples that had been in frozen storage for 12 h, 24 h, and 36 h and defrosted at 25 °C were tested. Impedance spectroscopy measured in PBS solution was used as the reference for comparison. Biofunctionalized MNPs were used to concentrate histamine in the extracts from the three samples, and impedance changes were recorded by the electrochemistry system. The impedance amplitude was increased by about 1.41%, 3.19%, and 8.23% for extracts from samples in frozen storage for 12 h, 24 h, and 36 h, respectively. It was calculated that the histamine concentrations were 0.1 μM, 0.73 μM, and 45.58 μM, for frozen storage in 12 h, 24 h, and 36 h respectively. This demonstrated the potential applicability of this nanoporous alumina membrane based electrochemical biosensor with biofunctional MNPs concentration for histamine determination in fish.

## 4. Conclusions 

In summary, a nanoporous alumina membrane based electrochemical biosensor with MNPs has been successfully developed for target histamine concentration and detection. Biofunctionalized MNPs conjugated with anti-histamine antibody were used to concentrate histamine from samples to an anti-histamine antibody functionalized nanoporous alumina membrane and captured in the nanopores. The blocking effect was evaluated by impedance spectra across the nanoporous alumina membrane. By quantifying the impedance change, histamine can be detected with low concentrations and the LOD reached was as low as 3 nM. Compared to histamine detection without MNPs, nanoporous alumina membrane based electrochemical biosensor with MNPs presented high sensitivity for histamine detection. This biosensor can also be successfully applied for histamine determination in saury that was stored in various conditions, showing promising applications for various food quality assessment and safety support.
